# Efficiency of Chitosan Nanocarriers in Vaccinology for Mucosal Immunization

**DOI:** 10.3390/vaccines11081333

**Published:** 2023-08-06

**Authors:** Salvatore Calogero Gaglio, Massimiliano Perduca, Donato Zipeto, Giuseppe Bardi

**Affiliations:** 1 Department of Biotechnology, University of Verona, Cà Vignal 1, Strada Le Grazie 15, 37134 Verona, Italy; salvatorecalogero.gaglio@univr.it; 2 Department of Neurosciences, Biomedicine and Movement Sciences, University of Verona, Strada Le Grazie 8, 37134 Verona, Italy; 3 Nanobiointeractions & Nanodiagnostics, Istituto Italiano di Tecnologia, Via Morego 30, 16163 Genova, Italy

**Keywords:** mucosal barrier, chitosan nanoparticles, vaccines, mucosal vaccines, local immunization

## Abstract

The mucosal barrier constitutes a huge surface area, close to 40 m^2^ in humans, located mostly in the respiratory, gastrointestinal and urogenital tracts and ocular cavities. It plays a crucial role in tissue interactions with the microbiome, dietary antigens and other environmental materials. Effective vaccinations to achieve highly protective mucosal immunity are evolving strategies to counteract several serious diseases including tuberculosis, diphtheria, influenzae B, severe acute respiratory syndrome, Human Papilloma Virus infection and Acquired Immune Deficiency Syndrome. Interestingly, one of the reasons behind the rapid spread of severe acute respiratory syndrome coronavirus 2 variants has been the weakness of local immunization at the level of the respiratory mucosa. Mucosal vaccines can outperform parenteral vaccination as they specifically elicit protective mucosal immune responses blocking infection and transmission. In this scenario, chitosan-based nanovaccines are promising adjuvants-carrier systems that rely on the ability of chitosan to cross tight junctions and enhance particle uptake due to chitosan-specific mucoadhesive properties. Indeed, chitosan not only improves the adhesion of antigens to the mucosa promoting their absorption but also shows intrinsic immunostimulant abilities. Furthermore, by finely tuning the colloidal properties of chitosan, it can provide sustained antigen release to strongly activate the humoral defense. In the present review, we agnostically discuss the potential reasons why chitosan-based vaccine carriers, that efficiently elicit strong immune responses in experimental setups and in some pre-clinical/clinical studies, are still poorly considered for therapeutic formulations.

## 1. Introduction

The outbreak of the COVID-19 pandemic reminded us of the importance of vaccines in the fight against infectious diseases induced by emerging and re-emerging uncontrolled pathogens continuously worrying the World Heathy Organization (WHO) [1]. It is estimated that one-third of the world’s population is infected with *Mycobacterium tuberculosis* (*M. tuberculosis*) and that approximately 9.6 million may develop active tuberculosis each year resulting in approximately 1.5 million deaths [2]. In addition, almost 70% of all deaths in very young children are caused by infectious diseases, with the highest percentages due to infections of the mucosal membranes, such as pneumonia (18%) and diarrhea (15%) [3].

Interestingly, one of the reasons behind the rapid spread of severe acute respiratory syndrome coronavirus 2 (SARS-CoV-2) was the lack of previous immunity and the availability of mucosal immunization [4]. The latter was likely responsible for infections of vaccinated people who did not show disease symptoms. Mucosal membranes constitute a huge surface area of approximately 40 m^2^ in humans, predominantly in the respiratory, gastrointestinal and urogenital tracts, as well as eye cavities. They play a key role in the interactions with the microbiome, dietary antigens and several environmental materials [5]. Mucosae are considered hot spots where the chance of pathogen entry is greatest. Therefore, an effective vaccination to elicit highly protective local immunity in the mucosa is an emerging strategy to counteract severe infectious diseases including tuberculosis, diphtheria, influenzae B, severe acute respiratory syndrome (SARS), Human Papilloma Virus infection (HPV) and Acquired Immune Deficiency Syndrome (AIDS) [6,7,8,9,10,11]. In this regard, mucosal vaccination may outperform the parenteral route in eliciting local and specific immune responses capable of blocking infection or transmission [5]. Among the materials used to develop mucosal vaccines, the natural polymer chitosan has shown promising features as a delivery system, in particular, its mucoadhesive [12] and intrinsic immunostimulant properties [13]. Therefore, chitosan can be used as an advantageous delivery system also acting as an adjuvant to provide sustained vaccine release and strongly activate the local defense system.

In nanomedicine, several well-described facilitations of specific drug delivery through mucosal barriers by chitosan nanoparticles (NPs) have been reported. For instance, intestinal adsorption of doxorubicin and tamoxifen was improved by encapsulating these drugs in chitosan NPs [14,15]. Furthermore, brain targeting and increased bioavailability of carbamazepine were achieved by using chitosan-based NPs as nasal carriers [16]. In addition, the positive charge of the polymer is another feature widely exploited to design chitosan NPs suitable for gene and antiviral-drug delivery [17,18].

In this review, we focus our attention in particular on the efficacy in vaccinology of chitosan-based carriers for the mucosa and its potential and limitations for future vaccine formulations.

## 2. Mucosal Structure

The mucous membrane, known as mucosa, is the soft tissue covering the body’s channels and organs in the digestive, respiratory and reproductive systems. The mucosa constitutes a huge surface area in humans, allowing various interactions with the microbiome, dietary antigens and diverse environmental materials. The structure of the mucosa presents several physiological barriers that interfere with the uptake of vaccine antigens and determine their rapid clearance. These barriers are determined by the cellular structure of epithelial surfaces (i.e., tight junctions), the presence of enzymatic barriers (i.e., nucleases and proteases) and the mucus. The latter creates a gelatinous layer which, due to its sticky nature and rapid turnover, prevents the entry of potentially dangerous substances or infectious agents. At the same time, the mucus inhibits the diffusion of NPs linked to drugs or vaccine antigens, thereby greatly reducing their efficacy. As the first barrier against pathogens, it also exerts critical roles in immunity and several physiological processes, such as the absorption of nutrients in the gastrointestinal tract.

**Figure 1:**
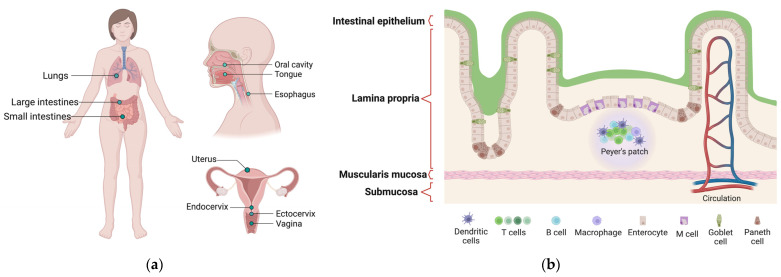
(**a**) Mucosal tissue of the human body. (**b**) The architecture of the small intestinal mucosa as an example of the tissue structure.

The mucosa is a complex structure consisting of three different overlapping layers (Figure 1) [19]:

- The epithelial layer is the most superficial. Epithelial cells secrete a thick, gel-like mucus that protects the body from irritants and gives the mucous membrane its name. There may be one or more layers of cells and they may be stacked in columns or arranged like bricks. Epithelial cells also have a high turnover rate, frequently replacing each other to clear out invasive particles. Some cells in this layer have small complex structures called cilia, which help to remove extraneous substances.
- The lamina layer is a connective tissue to which the epithelium attaches, and it is considered the middle layer of the mucosa. The lamina is composed of structural protein molecules, nerves and veins. It thus plays a crucial role in blood supply to the epithelium, keeping cells in place and binding them to the underlying smooth muscle. Its nerves respond to muscle fluctuations to change the shape of the epithelium as needed. Several varieties of immune cells are present in this layer seeking out and destroying intrusive microbes.
- The deeper layer is named muscularis mucosae, as it is mainly composed of smooth muscles. The muscularis mucosae provides a perpetual motor function that keeps the mucosa moving. This dynamic feature helps the lining to stretch and contract along with the various organs of the digestive system during their activity. In addition, it helps the mucous membrane to perform cleaning functions by keeping the hair-like cilia moving on the surface layer cells.

The intricate and complex structure of the mucosa poses a challenge for the development of mucosal vaccines: the delivery of antigens must be able to overcome the mucus layer present in the epithelium in order to achieve sufficient quantities to effectively activate adaptive immunity.

## 3. Needle-Free Vaccines and Mucosal Vaccination

Vaccines were developed to provide direct protection to immunized individuals through B and T cell-dependent mechanisms. Two crucial “players” that define vaccine performance are certainly the type of antigen and the adjuvant. However, the route of administration also has a strong influence on efficacy, along with the ability to be unaffected by storage temperature variations [20]. Depending on the site of entry, the vaccine encounters different types of “antigen presenting cells” (APCs), such as dendritic cells or macrophages [21]. APCs expose antigens on their surface and present them to lymphocytes, the effector cells of the adaptive immune system. The way the antigen is presented also determines the quality of the response in terms of efficacy, cellular memory, prolonged response and the specific cell subset involved.

Therefore, the antigen, adjuvant and route of administration must be chosen for each type of vaccine to optimize the induced immunological response [22,23]. While mandatory vaccines (i.e., anti-polio, anti-diphtheria, anti-tetanus, anti-hepatitis B, etc.) are injected into muscles, different needle-free vaccination systems using alternative routes of administration (i.e., oral, nasal, urogenital) also exist or are being developed [24].

Upon vaccine injection, the immune reaction causes a systemic effect. The antigen encounters the cells of the immune system, and the presence of an adjuvant causes a slow and continuous release depot (depot effect). For vaccines containing adjuvants, intramuscular injection is preferred because it reduces the risk of local skin reactions. Although, paradoxically, in remote areas with limited sterile conditions, injections aimed at inducing an immune response could be risky, providing a route of entry (i.e., the lesion caused by the syringe) for unexpected external pathogens. However, data show that protection against respiratory and sexually transmitted pathogens from locally administered vaccines induces greater protection than systemically/parenterally administered vaccines [25]. In general, injectable vaccines induce high antibody production but a lower T lymphocyte-mediated cellular response [21] and weaker induction of mucosal immunity [26]. For instance, intranasal vaccines are better suited to stimulate mucosal-specific immunity [27] as they are able to elicit the immune defense mechanism in situ.

The advantages and disadvantages of “common” injectable vaccines versus needle-free mucosal ones are summarized in Table 1.

**Figure 2:**
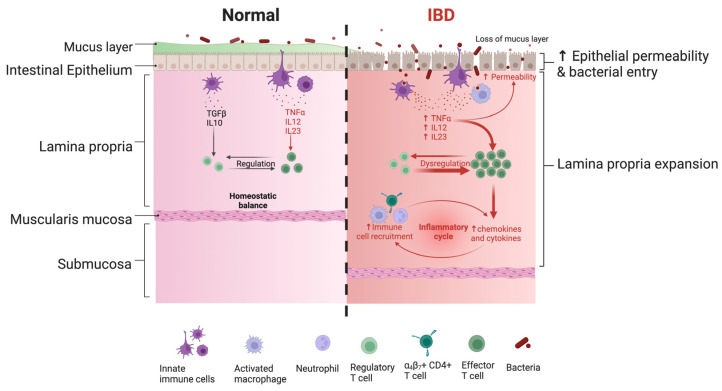
Schematic drawing of the immune response regulation in normal tissue and during inflammatory bowel disease (IBD) as an example of the pathological condition. The arrow (↑) means: “upregulation” of cytokines and “increased” permeability of the epithelium.

Mucous membranes are the hot spots of contact between the organism and the external environment, including potential pathogens facing a tightly regulated and specific mucosal immunity (Figure 2).

Unlike injected vaccines, mucosal vaccines are captured by innate immune cells patrolling the lamina propria close to the epithelium (Figure 2). Mucosal vaccine internalization under physiological conditions allows activation of the adaptive response without any injury to the tissue. Although very limited, the injection induces local damage stimulating the dysregulated activation of effector cells similar to local pathological conditions, as exemplified schematically by the IBD on the right side of Figure 2.

**Figure 3:**
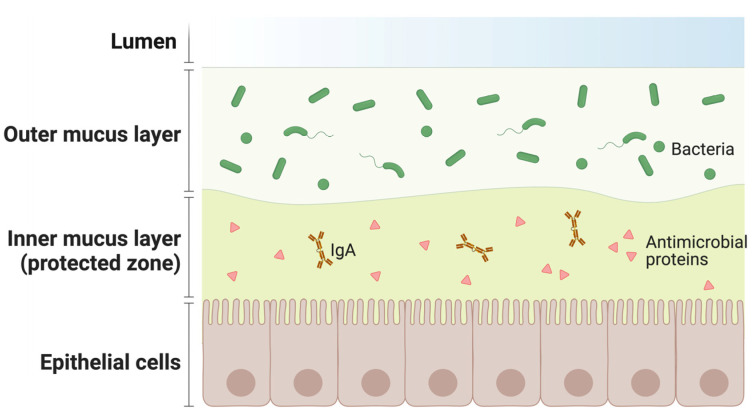
Organization of the mucosal defense barrier: the outer section of the mucus layer is in direct contact with the lumen and is exposed to external pathogens; the inner one, facing the epithelial cells, is rich in IgA and antimicrobial factors.

The mucosa is also particularly rich in secondary lymphoid organs harboring different immune cells producing antimicrobial proteins and B lymphocytes producing immunoglobulin A (IgA). These cellular configurations create a protective inner mucus layer (Figure 3).

Vaccine-activated B lymphocytes in the mucosa enrich the antimicrobial protein barrier of the inner mucus layer (Figure 3), increasing the efficiency of the “protected zone”. Moreover, IgA antibodies elicited by intramuscular vaccination differ from those induced by the compartmentalized, mucosal immune response to natural infection. Monomeric IgA present in plasma are less potent than the corresponding IgG, while dimeric IgA, the main type of antibody elicited at the mucosal level, are more potent against the same targets [30,31,32]. For instance, dimeric IgA deployed in mucosal secretions as the most effective antibody class able to neutralize air-borne pathogens, like SARS-CoV-2, in infected patients [27,29,33]. Interestingly, recent studies have shown that parenteral vaccines used against SARS-CoV-2 induce a weak mucosal response, although IgG can be detected in saliva and nasal fluid. Similarly, IgA are elicited in plasma [32], but they were detected at very low levels in saliva. This suggests that systemic vaccination does not stimulate an immunoglobulin response at the mucosal level, leading to reduced protection and a rapid decrease in the defense from infection in vaccinated individuals [34].

It is worth mentioning that in some cases, comparable results have been obtained between systemic and mucosal immunization. Nevertheless, the latter is often a safe, effective and less invasive alternative to parenteral vaccination [25].

## 4. Mucosal Immunity and Immunization

Mucosal vaccines could outperform parenteral vaccination in blocking infection and transmission by providing robust protective immune responses in the sites of pathogen penetration [7,9,35]. The induction of specific adaptive immunity in the mucosal first line of defense has the ability to prevent the onset of infection, rather than limiting the development of disease symptoms, making mucosal vaccines important tools for epidemic spreading [36].

As we previously mentioned, the predominant Ig isotype in the mucosal epithelium is the secretory IgA (SIgA) [37]. SIgA antibodies are able to neutralize toxins or pathogens in the mucosa through three different pathways: immune exclusion, antigen excretion and intracellular neutralization. In addition, they exert higher neutralizing activity than IgG due to their extracellular immune exclusion effect. After vaccination, it is desirable to achieve a sterilizing condition to prevent the spread of infection, which can be particularly dangerous in hospital settings during the management of frail patients [37,38,39].

Tissue-resident memory T (_TRM_T) cells also participate in the activation of adaptive immunity in the mucosa: indeed, they confer a rapid re-stimulation response that induces the production of inflammatory cytokines mediating tissue resistance and chemokines recruiting immune helper cells [40]. _TRM_T cells have been found at several mucosal sites exerting a decisive role in rapid responses to infections [41,42]. For example, lung-resident T and memory T cells play an essential role in fighting frontline viral and bacterial pathogens through direct mechanisms and by coordinating the adaptive immune system through the crosstalk pathway [43].

Therefore, mucosal immunization can trigger extensive adaptive IgA- and _TRM_T-mediated responses, which are, for instance, crucial for the establishment of protective lung immunity against tuberculosis [44]. Furthermore, strong cellular and humoral immune responses in the mucosa have the potential to induce sterilizing immunity by preventing pathogen binding and uptake across epithelial surfaces and, eventually, greatly reducing the risk of asymptomatic or paucisymptomatic infections. As the recent COVID-19 pandemic has taught, asymptomatic individuals are often the most difficult to intercept and heavily contribute to the diffusion of the virus and consequently to the pandemic risk [33].

## 5. Chitosan in Mucosal Vaccine Design

The adaptive immune response in the mucosa relies on the secretion of IgA and the activation of tissue-resident memory T cells. A mucosal vaccine should be able to efficiently deliver its cargo in order to trigger the secretion of antibodies penetrating the lamina layer and activate _TRM_T lymphocytes by continuous and prolonged exposure to the antigen. Furthermore, the rapid clearance by cilia and enzymes degradation by nucleases/proteases should be avoided. Nanotechnologies could overcome these obstacles by providing protection to the antigen, enhancing cellular uptake and increasing the retention time.

Often, the vaccine-active molecule is not sufficiently immunogenic to ensure a complete response. This requires stimulators (adjuvants) to successfully promote the immunization process. Chitosan-based nanomaterials have attracted great attention as adjuvants and delivery systems able to effectively boost mucosal immune responses [13,45].

To better understand the advantages of chitosan-based NPs, it is worth first recalling the chemical properties of the chitosan polymer and its valuable manipulation at the nano-scale. Chitosan is a biopolymer consisting of randomly distributed N-acetylated and deacetylated glucosamines [46,47]. It is produced by the deacetylation process of chitin [48,49,50], which is a component of the exoskeleton of shrimps, crabs and fungi. Interestingly, chitosan effectively fits into the circular economy as a high-added value material obtained from the waste product of chitin. In addition, chitosan is widely regarded as a nontoxic and hypoallergenic material suitable for use in medical and pharmaceutical applications [51].

**Figure 4:**
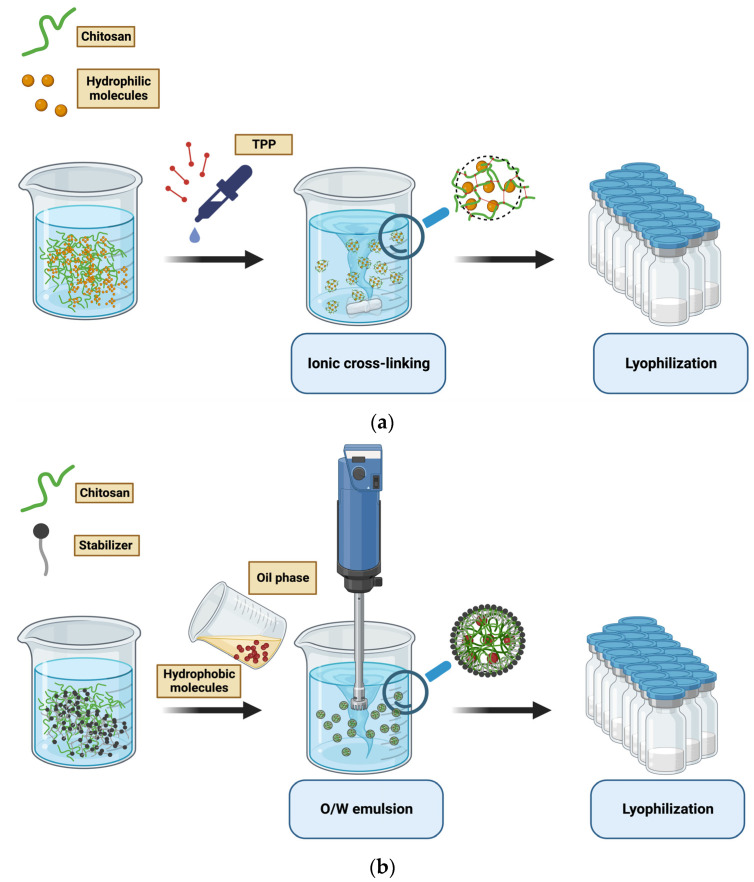
(**a**) Schematic illustration of chitosan NPs preparation by ionic cross-linking method; (**b**) schematic illustration of chitosan NPs preparation by O/W emulsion method.

The ionic cross-linking of chitosan NPs is the most common chitosan-nanomaterial preparation method. It is based on the interaction between a cross-linking agent and the amine or hydroxyl groups of chitosan (Figure 4a,b) [49]. Additionally, NPs are spontaneously formed in the process of polyelectrolyte complexation by adding nuclide acids to a chitosan acetic acid solution.

The reverse-micelle preparation method could also be used to prepare chitosan NPs of small size. This synthesis consists of dropping a polymer aqueous solution into an organic phase containing a surfactant with constant agitation to allow the formation of reverse micelles.

The described methods are suitable for loading hydrophilic molecules, while oil-in-water emulsion is used to produce 200–500 nm NPs embedded with hydrophobic molecules. An oil-in-water (O/W) emulsion is prepared by mixing an aqueous solution of stabilizer and chitosan with an organic solvent under mechanical shaking. Precipitation is the driving force for the nanostructure arrangement. Finally, NPs are collected and purified from solvent residues [18,49,52].

In vaccinology, chitosan mucoadhesive properties make this biopolymer suitable for the design of mucosal vaccines. Indeed, several studies have demonstrated the potential use of chitosan NPs and derivatives as promising vehicles for vaccine delivery, due to the presence of numerous positive charges on its surface facilitating electrostatic interactions with the negative charges that characterize the sialic acid present in the mucus lining the epithelium [53]. The increased retention time of the loaded antigen [54,55,56] also makes antigen uptake possible [57] by promoting permeability relying on its ability to open tight junctions between epithelial cells [58]. It is possible to further increase the permeability and diffusion capacity of chitosan nanoparticles by chemically modifying them by adding short tails of poly(ethylene glycol), poly(2-hydroxyethyl acrylate), poly(2-ethyl-2-oxazoline) or poly(N-vinyl pyrrolidone) [59]. Thus, chitosan NPs can more effectively target the immune cell population in the lamina layer. These NPs can provide sustained antigen release to strongly activate the humoral defense system by adjusting the colloidal properties. The immune triggering mechanism of chitosan NPs is based on enhancing antigen uptake and inducing macrophages to secrete inflammatory factors regulating Th1/Th2 balance tailoring a specific immune response [46].

Nanoscale chitosan intrinsic immunostimulatory properties have been observed in different studies and are reviewed elsewhere [60]. For example, Nantachit et al. [61] demonstrated that in vitro stimulation of human nasal epithelial cells with trimethyl chitosan NPs embedded with EDIII-D3 stimulated the secretion of several proinflammatory, Th1 and Th2 cytokines as well as chemokines. Particles uptake by DCs also upregulated the maturation markers (CD80, CD83, CD86, and HLA-DR) of DCs.

## 6. Chitosan Outcome in SARS-CoV-2 Immunization

The SARS-CoV-2 pandemic has also become a worldwide problem due to the rapid spread of variants, which limits the effectiveness of current intramuscularly administrated vaccines. The rapid establishment of an immune barrier at the level of the respiratory mucous membranes would be essential to counter the future spread of newly born viral variants. Moreover, the use of nanocarriers with adjuvant properties would certainly be helpful to allow a complete adaptive response in the mucosa.

In this regard, Shao-Hua Zhuo et al. designed a chitosan-based nanovaccine for inhalation. Spike protein-loaded chitosan NPs ((S@CS)NPs) were prepared using polyelectrolyte complexation. sIgA were detected in the bronchoalveolar fluid of mice immunized with (S@CS)NPs, whereas free Spike failed to elicit a sIgA response. In addition, SARS-CoV-2 Spike-specific IgG were also stimulated in bronchoalveolar liquid. Intriguingly, the IgG_1_/IgG_2a_ ratio in serum samples suggested that the intranasally administrated nanovaccine enhanced Th2 immunity. The authors highlighted the active role of (S@CS) in enhancing T cell recognition and activation due to the dual role of chitosan as an adjuvant and transporter. Additionally, memory T cells were detected in splenocytes after the third dose. Thus, S@CS challenge enhanced the production of CD4^+^ T cells and memory effector cells (CD3^+^ CD4^+^, CD44^+^ and CD62L^−^) [62]. The potential of mucosal chitosan-based nanovaccines to elicited not only a local mucosal immunization but also a systemic response needs to be further explored.

## 7. Modified Chitosan Nanocarriers and Pre-Clinical Trials

**Figure 5:**
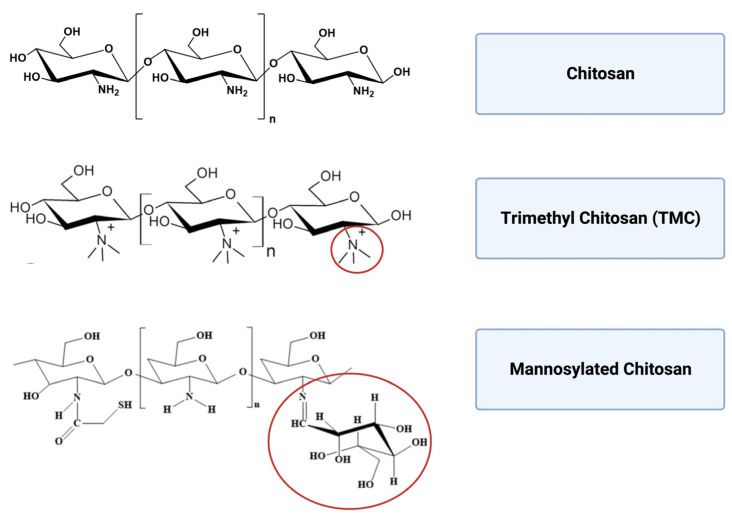
Molecular structure of chitosan, trimethyl chitosan and mannosylated chitosan.

To design efficient carrier-adjuvant material for different administration routes, chitosan is often chemically modified (Figure 5) and prepared in different versions, including microparticles, NPs and hydrogels. Microparticles are suitable for sustained release and oral administration, whereas NPs optimize cellular interaction and can be preferred for nasal administration. Chitosan hydrogel can increase retention time depending on the target tissue and the specific formulation. The biopolymer can be easily prepared under mild conditions (such as ionic gelation) and functionalized by surface chemistry modulation.

In the following subchapters, the different chitosan modifications are also described in terms of employment in pre-clinical tests, and the data are summarized in Table 2.

### 7.1. Trimethyl Chitosan

Trimethyl chitosan (TMC) shows a higher aqueous solubility and stability over a wide range of ionic conditions than the unmodified polymer. Furthermore, the modification does not affect the mucoadhesive properties and it allows improved crossing through the tight junctions of epithelial cells [54]. Because TMC-based NPs are smaller in size than unmodified chitosan NPs, they also show increased cellular uptake. The modified release pattern, higher loading efficiency and stronger positive surface potential make TMC-based NPs very attractive for the design of high-performance mucosal vaccines [63].

The potential of ovalbumin-conjugated N-trimethylaminoethylmethacrylate chitosan NPs (OVA-NP) in eliciting mucosal immune responses after nasal administration has been demonstrated in animal models by in vivo assays. Interestingly, the mice group treated with nasally administered OVA-NPs showed a 5.3-fold higher lymph node targeting index (LNTI) than the group receiving an intramuscular injection. When OVA was administered intranasally alone, it did not induce strong immune responses in the nasal cavity because of its low immunogenicity. Similarly, the injection of alum-precipitated OVA failed to induce mucosal immune responses. On the contrary, TMC-conjugation dramatically elicited sIgA levels in the mucosa [64].

These findings suggest the potential advantages of nasal vaccination in the prevention of respiratory infectious diseases by using a TMC-based carrier to enhance mucosal immune response. Other studies have demonstrated the ability of TMC-based carriers to elicit strong immunization by penetrating the mucosal epithelium and increasing the likelihood of boosting the antigen-presenting cell mechanism [54,65,66].

Upregulation of the B lymphocyte-mediated response has been also demonstrated by TMC NPs loaded with bacterial toxins. Tetanus toxoid-loaded TMC NPs (TMC(TT) NPs) were administered nasally in BALB/c mice. After the administration, antibody production against the tetanus toxoid antigen was detected. Unlike intramuscular tetanus toxoid injection, TMC(TT) NPs enhanced IgA secretion in saliva and IgG in blood serum, which significantly increased over the following 42 days [67].

In search of easy-to-use vaccines for tropical infectious diseases, TMC NPs embedded with domain III of dengue serotype-3 E protein (EDIII-D3) were prepared. The ability to stimulate human nasal epithelial cells by EDIII-D3 TMC NPs was proven, and the potential nanoformulated vaccine increased the secretion of several proinflammatory cytokines and chemokines. NP internalization by dendritic cells also upregulated the maturation markers (CD80, CD83, CD86 and HLA-DR) of DCs [61]. Antigen presentation-mediated overexpression of inflammatory mediators underlines the high potential of nasally administered TMC-based nanovaccines.

Further confirmation of TMC NPs’ enhanced cellular-mediated mucosal adaptive response has been shown by their use as mucosal immunopotentiators against Hepatitis B virus (HBV). TMC NPs embedded with an HBV surface antigen induced T cell proliferation and higher serum and nasal antibody titers than free antigen [68].

Efficient carriers are characterized by efficient protection of drug/nucleic acid load and its effective release on site. It has been proven that TMC encapsulation can also provide gastroprotection to the cargo emphasizing TMC NP suitability for oral administration. Actually, TMC(Omp31) NPs elicited a Th1–Th17 immune response. Moreover, when mice were challenged with *B. melitensis* 16 M, those orally vaccinated with TMC(Omp31) NPs were more protected than those immunized intraperitoneally [69].

Efficacy in mucosal vaccinology was also evaluated on chickens challenged with Newcastle disease virus (NDV). Compared with the commercial live attenuated NDV vaccine, chicken treated with O-2ʹ-HACC/pFDNA NPs induced higher production of anti-NDV IgG and sIgA antibodies and increased lymphocyte proliferation. Higher levels of IL-2, IL-4, IFN-γ, CD4+ and CD8+ T lymphocytes were also recorded exhibiting a complete immune response [70].

### 7.2. Mannosylated Chitosan

A synergy between specific humoral response and cellular immunity in the lung airways would be crucial for vaccine protection against tuberculosis infection. In fact, IgA could directly block the entry of *Mycobacterial tuberculosis* and modulate pro-inflammatory responses. Conjugation of mannose to chitosan (MCS) is another valuable strategy to enhance chitosan NP uptake by APCs [71]. In fact, mannose receptors are mainly expressed on macrophages and dendritic cell membranes to enable recognition and endocytosis of mannose-enriched pathogens [72].

An interesting hint for a notable application of mannosylated chitosan has been published by Manli Wu et al., who designed an MCS-DNA vaccine that could dramatically increase SIgA production in C57BL mice, contributing to the significant reduction of bacterial CFU in the lung. Adaptive poly-functional CD4^+^/CD8^+^ T responses were also detected [73].

### 7.3. Chitosan Hydrogel

To overcome the rapid clearance of the antigens in mucosal vaccination through nasal administration, which is mainly due to natural defense systems such as cilia, James G. Bedford et al. developed a chitosan-based hydrogel. The hydrogel structure aims at improving antigen retention time and, therefore, the chances of interacting with the target immune cells [66,74]. Highly protective local immunity was observed after treatment with a chitosan hydrogel vaccine loaded with influenza virus peptides. Immunized mice showed significant protection when infected by the influenza virus. The prolonged antigen retention provided by the hydrogel increased the proliferation of tissue-resident memory CD8^+^ T cells within the nasal mucosa [74]. Fascinatingly, these types of chitosan-based hydrogel could be explored to elicit nasal _Trm_CD8^+^ activation towards SARS-CoV-2 and other clinically relevant respiratory viruses.

**Table 2:** Summary of modified chitosan nanocarriers present in pre-clinical trials.

Chitosan-Based Nanocarriers	Antigen	Application	Activity and Immune Response	Advantages and Disadvantages	Administration Route	Reference
**Trimethyl** **chitosan**	Ovalbumin	Prevention of respiratory infectious diseases	Eliciting sIgA levels in the mucosa	High water solubility and increased stability	Intranasal	[48,55,56,57]
		Penetration of the mucosal epithelium and boosting the antigen presentation	Preserving the mucoadhesive properties and favouring the tight junctions crossing of epithelial cells			
		Upregulation of B lymphocyte				
	Bacterial toxin	Prevention against Tetanus	IgA secretion in saliva and IgG were detected in blood serum	Decrease in nanoparticle size than non-modified chitosan	Intranasal	[58]
			Increasing the secretion of several proinflammatory cytokines and chemokines	Increased cellular uptake		
				Increased loading efficiency and stronger positive surface		
	Domain III of dengue serotype-3 E protein	Prevention of tropical infectious diseases	Increase the secretion of several proinflammatory cytokines and chemokines		Intranasal	[52]
			Up-regulation of maturation markers (CD80, CD83, CD86 and HLA-DR) for dendritic cells			
	Hepatitis B surface antigen	Immunization against Hepatitis B virus	Induced T cell proliferation and increased serum and nasal antibody titer		Intranasal	[59]
	Omp31	Protection against Brucellosis	Eliciting the Th1–Th17 immune response		Oral	[60]
	O-2ʹ-HACC/pFDNA	Complete protection to Newcastle disease virus	Massive production of anti-NDV IgG and sIgA antibodies and increased lymphocyte proliferation		Intranasal	[61]
			High levels of IL-2, IL-4, IFN-γ, CD4+ and CD8+ T lymphocytes			
**Mannosylated** **chitosan**	MSC-DNA	Protection against Mycobacterium tuberculosis	Increase SIgA production in C57BL mice	Enhance chitosan nanoparticle uptake by APCs	Intranasal	[62,64]
			Trigger a poly-functional CD4+/CD8+ T responses			
**Chitosan** **hydrogel**	Influenza virus peptides	Protection against Influenza virus	Increase the proliferation of tissue resident memory CD8+ T cells within the nasal mucosa	Improve the antigen retention time	Intranasal	[65]
			Confers high protective local immunity			

## 8. Chitosan-Based Vaccines in Clinical Trials

Despite chitosan showing promising evidence in eliciting strong mucosal immunization in experimental setups, clinical efforts to standardize evaluation criteria and protocols to assess its potential use in human mucosal vaccination are lacking. Thus, we refer to efficacy in terms of antibody production and cellular response resulting from different pre-clinical studies that occurred in the last twenty years. All data in the chapter are summarized in Table 3.

Several studies have demonstrated that intranasally administered vaccines provide combined systemic and secretory immunity even in other distant mucosal membranes, such as the lung and the genital tract [75,76]. Intranasal immunization is therefore of great interest for infections caused by pathogens acquired via the mucosa, like *C. diphtheriae* or sexually transmitted microbes. Volunteers treated with intranasal CRM_197_-chitosan showed an sIgA response, while for those treated with intramuscular injection, no antitoxin sIgA was collected in the lavage fluid. Only intranasal administration activated nose-associated lymphoid tissue (NALT) for the sIgA response. Indeed, after the second immunization, a local antitoxin sIgA response was observed in the nasal lavage fluid of the vaccinated nostril and in the circulating IgA antitoxin antibody secreting cells (ASCs). The immune response induced by CRM_197_-chitosan was greater than CRM_197_ alone_._ The authors outlined the role of chitosan in stimulating efficient mucosal activation. The bio-adhesive property of the polymer could counteract the mucociliary action by favoring the localization of the antigen inside the nose, allowing its prolonged absorption. Furthermore, chitosan-based delivery preserved the intact antigen structure without causing histological changes [77].

Other studies demonstrated the potential role of CRM_197_ loaded in chitosan NPs to prepare a mucosal diphtheria vaccine. After nasal administration, a strong increase in Th2-type responses was observed in volunteers and it correlated with protective levels of toxin-neutralizing antibodies [78].

To develop an inexpensive but effective needle-free vaccine against both *C N. meningitidis* and *diphtheria*, healthy volunteers were given either a single intramuscular injection of (MCP)-CRM197 conjugate vaccine in alum or two nasal insufflations of chitosan-based (MCP)-CRM197. Nasal insufflation was generally safe and showed a serum bactericidal antibody (SBA) titer after two nasal immunizations in naïve subjects comparable to parenteral immunization. Furthermore, a concentration of MCP-specific immunoglobulin G close to that obtained with parental administration was recorded in naïve subjects. Interestingly, an almost four-fold higher concentration was collected in previously parenterally immunized subjects, suggesting that nasal administration could also be used as a booster in a second vaccination. Finally, only MCP-specific sIgA were induced at the mucosal site. In this phase I trial, authors highlighted the role of chitosan-based vaccines in eliciting both systemic and mucosal immunization, particularly against *C N. meningitidis* [79].

Samer S. El-Kamary et al. investigated a chitosan-adjuvanted Norwalk virus-like particle (VLP) intranasal vaccine, which elicited mucosal dendritic cells to enhance the immune response locally and systemically. Two phase 1 studies were conducted in healthy subjects (18–49 years) with no vaccine-related serious adverse events. One dose was sufficient to activate antibody secreting cells, while two doses were required to achieve an increased serologic antibody response. The observed mucosal priming phenomenon was supported by the presence of high frequencies of IgA and IgG ASCs in peripheral blood. Authors highlighted the ability of chitosan-adjuvanted VLP vaccines to induce potent mucosal and systemic immune responses including effector cell activity at a distant site of infection such as the gastrointestinal tract. Moreover, intranasal immunization elicited circulating ASCs of VLP-specific IgA and IgG with different homing potentials. IgA-specific ASCs upregulated receptors capable of conferring their homing ability in both intestinal mucosa and peripheral lymphoid tissues, while IgG-ASCs expressed homing receptors that support the localization in peripheral lymphoid tissues. This study provided evidence for the use of a nasal vaccine to combat gastrointestinal infections by triggering mucosal immune responses at distant sites from the administration point, such as the gastrointestinal tract [80].

## 9. Conclusions

Chitosan features allow for nanoscale manipulation resulting in several chitosan/chitosan-modified NP preparations, and it has been experimentally demonstrated to facilitate a defined mucosal immune response. However, its application in vaccine formulations is still absent as major market production. Different issues could be raised for this limitation. Though chitosan is generally recognized as safe (GRAS) by the FDA, the potential pro-inflammatory chitosan-induced reactions (adjuvant activity) might discourage pharmaceutical companies to face unexpected side effects. Furthermore, despite the easy scalability of chitosan production, the chemical processes employed may be harmful to the environment due to the large amount of alkaline waste and organic material produced [81]. On the other hand, chitosan itself is a natural compound that is easily obtainable from natural bio-sources and wastes from the crustacean food industry as a low-cost biocompatible nanoparticle precursor [82]. The natural bio-source, however, reduces the possibility to obtain chitosan batches with identical chemico-physical characteristics [83], making standardization more complicated. This can affect the final properties of the nanocarrier for large-scale products, such as vaccines, less reproducible [84]. Additionally, the production of chitosan NPs with modified surfaces could be challenging as functionalization processes increase manufacturing costs.

Nevertheless, several experimental results, pre-clinical and clinical trials of effective chitosan-based nanomaterials for vaccine preparation, as well as their economic [85] and ecological advantages, may lead to possible reconsideration of this material for the production of broad-spectrum mucosal vaccines against different infectious diseases.

## Figures and Tables

**Table 1:** Comparison between systemic and mucosal vaccination. [22,26,28] *, [24,29] **, [27] ***.

Administration	Advantages	Disadvantages
Injectable vaccines	systemic effect * antibody response * depot effect * simple and rapid * intradermal immunogenicity *	weak cell-mediated response * weak mucosal immunity *** need for a specialized operator ** maintenance of the cold chain **
Needle-free/mucosal	mucosal immunity *** tolerability and safety ** low costs ** ease of production, storage and distribution ** antigen stability ** ease of administration **	immunogenic tolerance * barriers in the gastrointestinal tract (pH, proteolytic enzymes) * not suitable for all types of antigens * most are preliminary **

**Table 3:** Summary of modified chitosan nanocarriers present in clinical trials.

Chitosan-Based Nanocarriers	Antigen	Application	Activity and Immune Response	Advantages and Disadvantages	Administration Route	Reference
**CRM_197_**-**Chitosan**	CRM_197_	Protection against Diphtheria	sIgA response	Efficient mucosal activation	Intranasal	[68,69]
			Activation of nose-associated lymphoid tissue	Preserving the antigen structure without histological changes		
			Presence of local antitoxin sIgA in the nasal mucus and in the circulating IgA antitoxin antibody secreting cells	Bio-adhesive property counteracts the mucociliary action, favoring the localization of the antigen inside the nose and allowing for prolonged absorption		
			Strong increase in Th2-type responses, correlated with protective levels of toxin-neutralizing antibodies			
(**MCP**)-**CRM197** **Chitosan**	(MCP)-CRM197 conjugated	Protection against both C N. meningitidis and diphtheriae	Serum bactericidal antibody titer after two nasal immunizations	Improve the antigen retention time	Intranasal	[70]
			Immunoglobulin G levels close to the ones obtained with parenteral administration			
**VLP**-**adjuvanted** **chitosan**	Norwalk virus-like particle	Protection against Norwalk virus	Elicited mucosal dendritic cells to enhance the immune response locally and systemically	Triggering the mucosal priming phenomenon	Intranasal	[71]
			Immunoglobulin G levels close to the ones obtained with parenteral administration	Inducing potent mucosal and systemic immune responses		
			High levels of IgA and IgG ASCs in peripheral blood	Inducing mucosal immune responses at distant sites from the administration point, such as the gastrointestinal tract		
			Presence of circulating ASCs of VLP-specific IgA and IgG with different homing potentials			

## Data Availability

Not applicable.

## Abbreviations

AIDS	Acquired Immune Deficiency Syndrome
ASCs	Antibody secreting cells
APCs	Antigen presenting cells
AUC0→4 h	Area under the concentration time-curve
DC	Dendritic cells
EDIII-D3	Domain III of dengue serotype-3 E protein
FDA	Food and Drug Administration
GRAS	Generally recognized as safe
HBV	Hepatitis B virus
HLA-DR	Human Leukocyte Antigen–DR isotype
HPV	Human Papilloma Virus infection
IgA	Immunoglobulin A
IBD	Inflammatory bowel disease
LNTI	Lymph node targeting index
MCS	Mannosylated chitosan
NPs	Nanoparticles
NDV	Newcastle disease virus
NALT	Nose-associated lymphoid tissue
O/W	Oil in water
OVA-NP	Ovalbumin-conjugated N-trimethylaminoethylmethacrylate chitosan NPs
SIgA	Secretory IgA
SBA	Serum bactericidal antibody
SARS	Severe acute respiratory syndrome
SARS-CoV-2	Severe acute respiratory syndrome coronavirus 2
(S@CS)NPs	Spike protein-loaded chitosan NPs
TMC(TT) NPs	Tetanus toxoid loaded TMC NPs
TRMT	Tissue-resident memory T
TMC	Trimethyl chitosan
VLP	Virus-like particle
WHO	World Health Organization
